# Screening to Detect Precursor Lesions of Pancreatic Adenocarcinoma in High-risk Individuals: A Single-center Experience

**DOI:** 10.5041/RMMJ.10353

**Published:** 2018-10-04

**Authors:** Jesse Lachter, Carly Rosenberg, Tomer Hananiya, Iyad Khamaysi, Amir Klein, Kamel Yassin, Elizabeth Half

**Affiliations:** 1 Department of Gastroenterology, Rambam Health Care Campus, Haifa, Israel; 2 The Ruth & Bruce Rappaport Faculty of Medicine, Technion–Israel Institute of Technology, Haifa, Israel

**Keywords:** Endoscopic ultrasound, pancreatic cancer, precursor lesions, screening

## Abstract

**Objective of the work:**

Pancreatic cancer (PC) is a deadly disease that is most commonly diagnosed at an incurable stage. Early diagnosis is the most important factor for improving prognosis. Evidence is beginning to accumulate that screening and surveillance may lead to the early detection of precursor lesions and/or pancreatic cancer in asymptomatic individuals. Proper screening methods and identification of such precursor lesions may enable effective pre-emptive interventions to prevent further fatalities. The primary objective of this project was to examine the feasibility of identifying precursor or early cancerous lesions in high-risk individuals by endoscopic ultrasound (EUS) screening to prevent the deaths from pancreatic cancer.

**Research aim:**

Pancreatic cancer screening guidelines, based on consensus opinions, have been applied in various tertiary centers around the world; however, evidence for effectiveness is lacking. At Rambam Health Care Campus, we have established a cohort of high-risk individuals, and we report our local 10-year experience results of screening for pancreatic cancer.

**Methods:**

Between 2008 and 2018, a cohort of 123 asymptomatic high-risk individuals came for annual/biannual EUS screening for pancreatic cancer. Retrospective and prospectively collected data were obtained, analyzed, and compared on the basis of several variables. These variables include age at beginning of screening, gender, smoking, obesity, diabetes, and presence of tumor markers, as well as the patients’ personal and family history of cancers. Findings on each EUS are described.

**Results:**

Three patients out of 123 underwent potentially life-saving surgery as a result of this screening program. All of these three had only one first-degree relative (FDR) with pancreatic cancer at the time of their first screenings, but two eventually had a second FDR with PC. Findings from 296 EUS exams regarding smoking, obesity, and other risk factors are presented. Minor, possibly trivial, EUS findings are found to be common. Detection of precursor pancreatic lesions is feasible with EUS screenings.

**Conclusions:**

Adherence was an important limiting factor in screening. Better stratification of patients according to specific risk factors, including thorough genetics and family history, may direct when and how to initiate screening. International collaborations, such as the International Cancer of Pancreas Screening (CAPS) Consortium, of which Rambam is a collaborating partner, are needed to collate evidence for impact of screening to prevent pancreatic cancer morbidity and mortality, and are essential to achieve proof of concept. Different countries with varying health-care systems and budgets can find variance of appropriateness of screening procedures.

## INTRODUCTION

Pancreatic adenocarcinoma is a merciless, usually fatal, disease. It is the third most deadly cancer in the US and Israel as well. The best hope for improvement of survival is prevention or very early detection.^1^ Primary prevention methods, such as cessation of smoking, reduction of alcohol intake, and proper exercise and dietary regimens, have been historically difficult to implement. Focused prevention methods, including detection of precursor lesions or early-stage pancreatic cancer (PC), might contribute to the prevention of this deadly disease. Certain genetic syndromes are associated with a high risk of PC, and screening has become a relatively new strategy for familial pancreatic cancer. The Pancreatic Cancer Research Center at Johns Hopkins, and many others, has shown that screening with endoscopic ultrasound (EUS) and/or abdominal imaging tests such as high-resolution computed tomography (HRCT) and magnetic resonance imaging (MRI) can detect a relatively high number of significant pancreatic neoplasms (7%–18%) in asymptomatic high-risk individuals with an inherited predisposition for pancreatic ductal adenocarcinoma.^2^

Although the majority of cases of PC are thought to be sporadic, it is estimated that 5%–15% of cases are caused by inherited genetic mutations.^3^,^4^ In this regard, two distinct high-risk groups were identified. The first group consists of high-risk individuals in whom PC develops within the setting of a well-defined cancer susceptibility syndrome or inherited disease. Numerous germline mutations, leading to an increased risk for developing pancreatic cancer, have been discovered.^5^–^14^ They are listed in Table 1.

**Table 1:** Cancer Susceptibility Syndromes or Inherited Disease with a Known Elevated Risk of Developing Pancreatic Cancer.

Syndrome	Gene(s)	Risk of Pancreatic Cancer
Hereditary breast and ovarian cancer (HBOC)	BRCA1 BRCA2	RR 2–3 RR 3–10
Familial cutaneous malignant melanoma (familial CMM)	CDKN2A (p16)	RR 8–45
Hereditary pancreatitis	PRSS1	SIR 60–90
Hereditary non-polyposis colorectal cancer (Lynch syndrome)	MLH1/MSH2/MSH6	RR 9
Peutz-Jeghers syndrome	STK11/LKB1	RR 75–135
Familial adenomatous polyposis (FAP)	APC	RR 4.5
Li-Fraumeni syndrome	p53	RR 7.5

Taken from Canto et al.,^15^ Copyright © 2013, BMJ Publishing Group Ltd and the British Society of Gastroenterology, with permission.

RR, relative risk; SIR, standardized incidence ratio.

The second and largest group consists of families with a strong family history of pancreatic cancer (two or three family members), despite lacking a known cancer susceptibility mutation or inherited syndrome. This condition is referred to as familial pancreatic cancer. Members of familial pancreatic cancer kindreds collectively have an increased risk of PC. Individuals with one FDR have a 4.6-fold increased risk, whereas persons with two first-degree relatives (FDRs) with PC have a 6.4-fold increased risk, and those with three affected FDRs have a 32.0-fold increased risk of developing the disease.^16^ This risk increases further as the number of FDRs with PC increases. People at average risk (with no relatives with pancreatic cancer) have a lifetime risk of 1.5% for PC.^16^

Screening of asymptomatic individuals aims to detect an early stage of non-symptomatic PC or, even more preferably, an advanced precursor lesion. Similar to the adenoma-carcinoma sequence in colorectal cancer, PC evolves through non-invasive precursor lesions. Known precursor lesions for pancreatic cancer are pancreatic intraepithelial neoplasia (PanIN), intraductal papillary mucinous neoplasms (IPMNs), and mucinous cystic neoplasms (MCNs).^17^ These precursor lesions are more common and of higher grade in patients with a strong family history of PC.^18^ In sporadic cases, it is estimated that a precursor neoplastic clone will take approximately 11–12 years to evolve into a malignant clone and an additional 7 years to develop metastatic subclones.^19^ Although it is unknown whether the progression of PC in hereditary cases follows the same pace compared to sporadic cases, these findings do seem to provide a window of opportunity to perform a timely intervention to prevent lesions from evolving into cancer. The premise of this strategy is that these precursor lesions can reliably be identified and stratified according to their risk of malignant transformation (e.g. degree of dysplasia) by a suitable surveillance technique.

The International Cancer of Pancreas Screening (CAPS) Consortium was formed in 2010 to help organize global PC screening and stimulate research to promote the development of evidence-based surveillance protocols (and included the first author of this report). As of 2018, more hospitals (currently 44 centers) from all over the world are linked to the CAPS Consortium, the Israeli site being at Rambam Health Care Campus. Small sample sizes are too small alone to definitively determine whether screening of the pancreas leads to reduction in PC morbidity or mortality while outweighing costs and potential harms of screening. However, each center varies its protocols for screening according to local constraints. Together, data from many centers, pooled into one worldwide registry, may help assess recommendations for pancreatic screening more reliably.

## PATIENTS AND METHODS

This is a single-center, prospective cohort study. Consecutive consenting high-risk adults from Rambam gastroenterology who underwent screening for PC have been traced over a 10-year period (2008–2018).

The patients were obtained by a referring gastroenterologist or by self-referral.

Data for this interim report were extracted from the electronic medical record including demographics, medical and social history, family history of cancer, genetic testing, carbohydrate antigen 19-9 (CA19-9) serologies, screening findings, and cytology results. Data were stored and managed in a de-identified fashion.

All patients underwent at least one EUS screening examination. Inclusion criteria were based on the CAPS criteria. In addition, another five inclusion criteria qualify the patients for screening: one FDR with PC, one second-degree relative with PC (both upon patient request), BRCA1 or 2 with one relative with PC, BRCA-positive without family history of PC upon patient request, and chronic pancreatitis. If there is a positive family history, patients are additionally referred for genetic counseling and appropriate genetic testing, which is at their personal expense. Exclusion criteria consisted of the following: age 75+, physically unfit for EUS screening, unable or unwilling to consent.

Special emphasis should clarify the inclusion criterion of having a single FDR. Our local decision was to compare the EUS exams to colonoscopy for colon cancer screening and prevention and early detection. Colonoscopy screening has been adapted in many countries to prevent a disease with a 5%–6% lifetime risk in average persons, with an average mortality of almost 40%. Pancreatic cancer with a single FDR has a 5% lifetime risk and carries a 95% mortality risk.^15^ Thus, although original CAPS criteria did not include EUS for persons with a single FDR, our center did include them. Notably, in a multinational survey by the expanding CAPS group, currently submitted for publication, significantly more centers are also justifying screening with similar, more liberal criteria (Canto M et al., manuscript submitted to British Journal of Surgery, unpublished paper, September 2018).

For patients at lowest risk with a normal EUS screening, a two-year follow-up period was recommended. This interval has received support from various centers for the youngest and least at-risk patients, at the annual discussions of the CAPS group. If a lesion was detected by EUS, recommendations were made for MRI or an earlier follow-up EUS depending on endoscopic evaluation. A fine-needle aspiration (FNA) was performed (EUS-FNA) at the decision of the endoscopist, based on standard clinical criteria.

The data herein were extracted from electronic medical records, including study eligibility: screening findings, the number of procedures performed, the outcome of procedures (if additional imaging was needed, surgery, family history, demographics, medical and social history, diabetes, tumor markers, and behavioral influences such as smoking, alcohol, and body mass index [BMI]). A data set was created in an Excel spreadsheet. Data were analyzed and stratified based on the screening indication, results of screening, and patient outcome. This single-center study was approved, and continues ongoing, by Rambam Health Care Campus institutional review board, which also performed a thorough on-site evaluation of the record-keeping and conforming to good clinical practice (GCP) of this study as part of randomly chosen assessments made by the academic center before the renewal of the permission to continue with the study in 2016.

## RESULTS

Altogether 123 patients were included in this study. All patients had at least one EUS performed (1–9 EUSs were performed per patient). Inclusion criteria for the patients, by indication, were as follows:

- Individuals with three or more affected blood relatives, with at least one FDR (*n*=8)
- Individuals with at least two affected FDRs with PC (*n*=9)
- Individuals with two or more affected blood relatives with PC, with at least one affected FDR (*n*=12)
- BRCA2 mutation carriers with one affected FDR (*n*=14)
- Mismatch repair gene mutation carriers (Lynch syndrome) with one affected FDR (*n*=3)

There were no cases included for the CAPS categories of having Peutz–Jeghers syndrome, P16 carriers with one affected FDR, BRCA2 mutation carriers with two affected family members (no FDR), and the category of PALB2 mutation carriers with one affected FDR.

The rest of the patients were documented as being screened due to family history of PC without necessarily specifying the number and specific relations of the affected family members.

In total, 296 EUSs were performed: 36 underwent just one EUS exam, 40/123 individuals (32.5%) had 2 EUSs performed; 24 (19.5%) had 3; 13 (10.5%) had 4; 7 (5.6%) had 5; 2 (1.6%) had 6; and 1 (0.8%) individual continued to 9 procedures. The study group comprised 70 males and 53 females, with an average age of 57 years. There were 69 (56%) non-smokers, 16 (13%) were current smokers, 16 (13%) had smoked in the past, and 22 (17.8%) patients had an unknown smoking status. Of the current smokers and past smokers in the study, 4 (25%) and 1 (6.25%) patients had abnormal findings on EUS, respectively, relative to 17 (24.6%) of the non-smokers (*P* = NS). Of the patients in this study, 7 (5.7%) were overweight (BMI 25–30), 18 (14.6%) were obese (BMI>30), and 39 (31.7%) patients did not have a recorded BMI. Five (27.7%) obese patients, 3 (42.8%) overweight patients, and 10 (16.9%) non-obese patients had abnormal results on screening (*P*=0.22).

Fourteen (11.3%) patients had non-insulin-dependent diabetes mellitus, 4 (3.2%) were borderline diabetics, 66 (53.6%) patients did not have diabetes, and 39 (31.7%) had unknown status. Four (3.25%) patients had elevated cancer antigen (CA) 19-9 levels; among them, only 1 had abnormal findings on EUS.

Genetic mutations were taken into account as well as a relevant family history of malignancies. A minority, 22.7% of the patients in this study, completed genetic testing (Table 2).

**Table 2:** Findings from Genetic Testing.

Variable	*n*
BRCA	14
BRCA1	3
BRCA2	9
Unspecified	2

MSH6	3

*n*, number of individuals

The two genetic mutations recorded were of BRCA1/2 and MSH6 causing Lynch syndrome. Fourteen patients tested positive for BRCA. Of those, 2 patients had abnormal EUS results on their second and third procedures. Both patients were BRCA2 carriers. The first patient was found to have a 3 mm side branch dilatation in the body of the pancreas on the second screening. A follow-up EUS revealed the same result one year later. The second patient was found to have a 5 mm round hypoechoic region in the pancreatic body/neck, on the third screening. This lesion was slightly hard on elastography.

Of the 3 Lynch syndrome patients (MSH6), 2 (66.6%) patients had abnormal findings on their EUS; the first patient had two pancreatic cysts, 3 mm in the head of the pancreas and 4.5 mm in the body, on the first screening. The patient was sent for an MRI with magnetic resonance cholangiopancreatography that yielded no further findings. On the following two EUS surveillances that he underwent, the patient’s pancreatic cysts have remained stable. The second patient had one small 2.6 mm pancreatic cyst found on the third screening. He was scheduled for a follow-up EUS in one year.

Of those with abnormal results on the first screening, 17 (47.0%) were cystic findings ranging from approximately 2 mm to 7 mm. Follow-up EUS was recommended. One patient was found to have a 51 mm cystic and solid septated lesion—suspected serous cystadenocarcinoma stage T3N0M0—in the head of the pancreas. The patient underwent a Whipple operation and was diagnosed with adenocarcinoma of the pancreas.

In other cases, pancreatic abnormalities were only detected on the second or third round of screening. Ten (25.0%) patients had abnormal findings on the second screening. Of these, 4 were completely new, consisting mainly of cysts and dilatations, 2 were progressive from the first screening (enlarging cysts), and the remaining 4 were stable and consistent with findings in the first EUS. Eleven (45.8%) patients had abnormal findings on the third screen. Among these findings, 5 were new, including lobulations and cysts, and in 2 cases IPMN lesions were found. Of these 2 patients, 1 patient was discovered with an advanced IPMN lesion and underwent Whipple operation. The pathology of the lesion revealed a neuroendocrine tumor (stage 1, grade 1). The other patient was discovered to have a 7 mm main branch IPMN—her two previous screenings were normal. A FNA was performed and demonstrated mucinous epithelial cells. She was scheduled for a further EUS in one year. One year later, the EUS was unchanged. One other IPMN was identified on a third EUS in a patient from which the second screening displayed a 4.5 mm cyst. The patient was seen one year later for a follow-up EUS, demonstrating no further changes.

During the process, there were no complications from any of the 296 EUS procedures.

## DISCUSSION

Cancer prevention is increasingly in vogue. Overall, PC screening programs in high-risk individuals, such as at Rambam Health Care Campus, have been shown to aid in raising awareness of risks as well as to carefully select patients to pursue the most appropriate screening procedure and intervals. In addition to the focus on PC risk, lifestyle measures with broader implications were addressed and documented in EUS reports. Patients were encouraged to reduce lifestyle risk factors, to stop smoking, strive for ideal weight, control their diabetes if present, and get regular and sufficient exercise.

Pancreatic cancer is a challenging field due to its asymptomatic nature until its final grueling and usually lethal outcome. Individuals at risk of familial pancreatic cancer are recommended to participate in prospective screening programs to detect precursor lesions or early-stage pancreatic cancer.^20^ The age at which screening should be initiated remains uncertain. Most screening programs begin around 10 years below the youngest age of onset in the family. In our study the average age at screening was at 57 years. The median age at diagnosis of pancreatic cancer in the general population is 72. Below the age of 50, the incidence of pancreatic cancer is much lower. The incidence, however, increases sharply by the age of 75–79.^20^ The findings of a study on the refinement of screening for familial pancreatic cancer^19^ indicated that screening of high-risk individuals for familial pancreatic cancer rarely reveals significant or potentially relevant pancreatic lesions before the age of 50; the authors recommended raising the screening age to improve efficacy of findings, reduce psychological distress of the patients, and reduce costs of familial pancreatic cancer screening.

Recommended screening methods are based on EUS and MRI. This is considered the best approach to detect solid pancreatic tumors and IPMNs ≤1 cm in size as well as irregularities of the pancreatic duct.^19^ These lesions are generally asymptomatic and thus difficult to diagnose. In one study,^20^ however, only detection and surgical treatment of T1N0M0 adenocarcinoma and the high-grade PanIN3, main-duct IPMN, and branch-duct IPMN with high-grade dysplasia were determined to be a success of screening.

In this study, patients who underwent a primary screen yielded 23.5% abnormal findings from all EUS baseline screenings. A larger proportion (45.8%) of abnormal results was seen on the third screening of patients. Of those, 40% were identified as IPMNs, allowing a window of opportunity to implement prevention of further progression. Based on our results, long-term adherence may be an important factor in discovering potentially harmful lesions. Although the present data are inconclusive, studies,^20^,^21^ have expanded the previous annual interval between screenings to biannual screening intervals in cases of patients with unremarkable pancreatic findings in baseline EUS screening. In our center, younger patients with no abnormality are usually scheduled at two-year intervals, whilst patients with any findings or patients as old as their closest FDR was at the time of getting pancreatic cancer are offered annual EUS exams. It is notable that the costs of EUS and genetic screening are quite variable from country to country, thus the cost for a quality-adjusted life-year saved would be very different. The cost for an out-of-pocket EUS examination in our hospital is the equivalent of US$350. Some centers in other countries (such as the USA) charge 10–30 times as much for an EUS exam. The local costs of MRI of the pancreas are about double that of EUS. Because the MRI studies are non-invasive, this adds to their attractiveness to some screenees. The Israeli public have universal health-care coverage, based on a nationally approved basket of included services. Currently, EUS and MRI screenings of the pancreas are not expenses covered by the basket and are paid out of pocket. However, in over 90% of the above 296 examinations, the health maintenance organizations did cover the complete costs of the EUS exams. It did not cover genetic tests.

Although EUS is not a harmless procedure, of these 296 procedures performed in our study, no complications occurred. However, there are serious risks which need to be considered. One study^22^ based in Israel, on the mortality risk of EUS, found an average of one death from each 2,500 procedures. In the cases analyzed, none resulted from FNA procedures. This study described 12 deaths from EUS procedures; 7 were local cases, and 5 cases were from outside Israel. Another study^23^ evaluated the morbidity and mortality of EUS-guided FNA from 51 papers with a total of 10,941 patients. They found that the overall rate of EUS-FNA specific morbidity was 0.98% (107/10,941). The complications found in EUS procedures consisted mainly of pancreatitis (33.64%) and post-procedure pain (34.58%). The mortality rate attributed to EUS-FNA morbidity was 0.02% (2/10,941). They concluded that EUS-FNA-related morbidity and mortality rates are relatively low, and most are associated with events of mild to moderate severity.

Three patients in total underwent surgery. Of these, two patients had findings discovered on the first screening, and one of them had findings discovered on the third EUS. Each is now discussed. One patient was screened due to having one FDR—his father had early pancreatic cancer. The patient was found to have a high-grade dysplastic ampullary tumor, which was removed by a duodenotomy without needing a full Whipple procedure. The second patient had a neuroendocrine tumor found by EUS and surgically removed, which may be considered to have been an “incidentaloma.” Her mother, and subsequent to her operation her brother, had pancreatic adenocarcinomas and died of these. None of the operated patients had major complications from their surgery, and all of them benefited. The patient found incidentally to have a 51 mm cystadenocarcinoma had what was considered to be an R0 resection.

One study^23^ explored the risk of surgery in the resection of high-risk pancreatic lesions. The study discussed two cases. Both patients were high-risk individuals for pancreatic cancer. Both patients had screen-detected lesions with very different outcomes. On one hand, it can be significantly beneficial and life-saving, but, on the other hand, screening and intervention can be risky and severely damaging. This study also demonstrated how one screening method alone may not be sufficient to detect or confirm pancreatic lesions. In our EUS-based study, every patient underwent EUS screening, while in the study by Wang et al., the methods of screening were dominated by MRI.

Screening is important and recommended in high-risk patients. However, much more evidence is needed to determine (1) when to start screening for each risk-profile, (2) how to manage screen-detected findings, and (3) if the benign nature of pancreatic findings can be determined in order to prevent potential surgical complications.

### Limitations

There were specific limitations to our study. Firstly, the study consisted of a small patient group with inconsistent, suboptimal adherence to screenings. Secondly, the inclusion criteria were broad (i.e. liberal) for the reasons stated above, and not all included individuals were considered high risk according to standard CAPS criteria, a fact that might be expected to have influenced the frequency of screen-detected lesions. A third limitation was based on the availability of data. Certain characteristics—such as extensive family history documentation that might be expected from a genetic counsellor, and history of smoking, obesity, and diabetes—were collected freehand rather than computer-tabulating boxes being checked, and as a result were commonly lacking in the electronic medical records data system; they were thus often not calculable. This made the correlation between patient characteristics and abnormal results on EUS screenings less inclusive. Most patients declined genetic screening due to the personal expense. This therefore restricted our results. Genetic testing prices have dramatically decreased in the past year, which has helped fuel a dramatic increase in patient acceptance and performance of genetic mutation analysis. Additionally, the shortage of EUS availability may have restricted the number of screenings that could have been performed. In 2017 just over 700 EUS examinations were performed in our tertiary hospital with a large associated oncology center. Most EUS examinees are outpatients who have waited 4–8 weeks for their appointments. The availability of doing screening exams for healthy individuals gets lower priority than for other indications. The long waiting time may cause some potential screenees to consider screening to be of low importance and thus choose to go without. We are also aware that some potential candidates for screening, after coming for an initial consultation, have gone to local centers closer to their homes to have EUS exams performed in the one other center in Israel now known to be doing prospective EUS research-based screening, and others have gone to non-academic centers to have EUS performed and became “lost to our follow-up.” This situation is not unique. In their most recent and very encouraging study on the impact of screening, Canto et al. reported that fully 40% of patients coming to Johns Hopkins at least once then went on to have any further screening done at other centers and thus were not included in the results of the EUS research on screening. Nonetheless, the very impressive increase of survival at their center, over 80% three-year survival in patients found to have PC in screening programs versus 8% amongst those not in screening, provides all centers active in screening with evidence-based and highly significant encouragement.^24^

## CONCLUSION

In conclusion, we have shown that PC screening in high-risk populations is feasible and has the potential to save lives. However, more evidence is needed regarding the age at which to initiate screening, intervals between screening, and the management of asymptomatic precursor lesions. By each center publishing its results, the information when pooled together may yield answers to these questions and may prevent progressive disease, especially for those in the highest-risk categories. Endoscopic ultrasound alone cannot be expected to be sufficient to prevent all cases of familial pancreatic cancer; however, the screening programs collectively may well save lives. Hence, continuing efforts to screen for and prevent pancreatic cancer are essential.

## Abbreviations

BMI: body mass index
CA: cancer antigen
CAPS: International Cancer of Pancreas Screening
EUS: endoscopic ultrasound
FDR: first-degree relative
FNA: fine-needle aspiration
HRCT: high-resolution computed tomography
IPMNs: intraductal papillary mucinous neoplasms
MCNs: mucinous cystic neoplasms
MRI: magnetic resonance imaging
PanIN: pancreatic intraepithelial neoplasia
PC: pancreatic cancer
